# Two new species from tree-hole humus in Guizhou Province, China: *Leptobacillium doupengense* (*Hypocreales*, *Cordycipitaceae*) and *Metapochonia doupengshanensis* (*Hypocreales*, *Clavicipitaceae*)

**DOI:** 10.3897/mycokeys.137.196582

**Published:** 2026-07-27

**Authors:** Wen-Kai Lin, Li-Li Lou, Xiao Zou, Ye-Ming Zhou

**Affiliations:** 1 Institute of Fungus Resources, Key laboratory of Plant Resource Conservation and Germplasm Innovation in Mountainous Region (Ministry of Education), College of Life Sciences, Guizhou University, Guiyang 550025, Guizhou, China Institute of Fungus Resources, Key laboratory of Plant Resource Conservation and Germplasm Innovation in Mountainous Region (Ministry of Education), College of Life Sciences, Guizhou University Guiyang China; 2 Guizhou Key Laboratory of Agricultural Microbiology, Guiyang, 550025, Guizhou, China Guizhou Key Laboratory of Agricultural Microbiology Guiyang China

**Keywords:** *Leptobacillium* spp., *Metapochonia* spp., morphology, phylogenetic analysis

## Abstract

Fungal strains were isolated from humus substrates collected from tree holes in Doupengshan Scenic Area, Duyun City, Guizhou Province, China, a region characterized by a typical karst ecosystem. Tree-hole humus represents a spatially restricted and nutrient-rich microhabitat formed by decomposed plant material and organic residues, yet hypocrealean fungi associated with this substrate remain poorly explored. Based on a combined five-gene dataset (ITS, nrSSU, nrLSU, *tef*-*1α* and *rpb1*), together with phylogenetic analyses and morphological comparisons, two new species of *Hypocreales* were identified: *Leptobacillium doupengense***sp. nov**. and *Metapochonia doupengshanensis***sp. nov**. Both species formed distinct lineages in the phylogenetic tree and were clearly separated from other species within their respective genera. This study provides detailed morphological descriptions and illustrations of *L. doupengense* and *M. doupengshanensis*, along with comparisons with related species in their genera. The discovery of these two species highlights the potential of tree-hole humus in karst ecosystems as an overlooked source of hypocrealean fungal diversity. These findings provide morphological and molecular evidence for recognizing and distinguishing *L. doupengense* and *M. doupengshanensis* and contribute additional taxonomic data to clarify species diversity and phylogenetic relationships within *Leptobacillium* and *Metapochonia*.

## Introduction

Zare and Gams (2016) proposed the genus *Leptobacillium* Zare & W. Gams during their revision of the *Albo-erecta* section of the former genus *Verticillium* Nees. Phylogenetically, this genus belongs to *Cordycipitaceae*. The generic name *Leptobacillium* is derived from its slender conidia. Zare and Gams (2016) originally designated *Leptobacillium leptobactrum* (W. Gams) Zare and W. Gams as the type species of this genus. This species comprises two varieties, L. leptobactrum var. leptobactrum and L. leptobactrum var. calidius (W. Gams) Zare and W. Gams, which are distinguished by their optimal growth temperatures. Species within the genus *Leptobacillium* possess two morphologically distinct types of conidia. The first type, which is ellipsoidal to subglobose, is primarily found at the apex of the conidial chain, whereas the second type, cylindrical, bacilliform, or fusiform, is distributed along the remainder of the conidial chain (Zare and Gams 2016; Leplat et al. 2022). To date, 14 species have been accepted in the genus *Leptobacillium*. They have been isolated not only from insects and fungi, but also from plants, rocks, freshwater environments, mural paintings, and decaying wood (Liu and Cai 2012; Zare and Gams 2016; Crous et al. 2018; Gomes et al. 2018; Sun et al. 2019; Okane et al. 2020; Leplat et al. 2022).

In contrast to *Leptobacillium*, *Metapochonia* Kepler, S.A. Rehner & Humber was established to accommodate several species previously placed in *Pochonia* Bat. & O.M. Fonseca. Kepler et al. (2014) transferred five species previously assigned to *Pochonia*, *P. bulbillosa* (W. Gams & Malla) Kepler, S.A. Rehner and Humber, *P. goniodes* (Drechsler) Kepler, S.A. Rehner and Humber, *P. microbactrospora* (W. Gams & Zare) Kepler, S.A. Rehner and Humber, *P. rubescens* (Zare, W. Gams & López-Llorca) Kepler, S.A. Rehner and Humber, and *P. suchlasporia* (W. Gams & Dackman) Kepler, S.A. Rehner and Humber, to the newly established genus *Metapochonia* based on a multi-locus phylogenetic analysis. This genus is morphologically very similar to *Pochonia*, as both produce dictyochlamydospores and are parasitic on nematode eggs or cysts (Zare and Gams 2007; Kepler et al. 2014). *Metapochonia* currently comprises 11 species, which have been isolated from soil, plant roots, the sclerotia of *Ophiocordyceps sinensis* (Berk.) G.H.Sung, J.M.Sung, Hywel-Jones and Spatafora, river water, and nematode eggs (Huang et al. 2015; Zhang et al. 2017; Labuda et al. 2018; Kondo et al. 2020; Crous et al. 2024).

Guizhou Province, China, is characterized by a typical karst ecosystem and heterogeneous microhabitats that provide favorable conditions for fungal diversity. Numerous hypocrealean fungi have been reported from this region, indicating that Guizhou represents an important area for exploring the diversity of *Hypocreales* (Wen et al. 2017; Chen et al. 2019, 2022d, 2025). However, previous studies have mainly focused on fungi isolated from host surfaces, fruiting bodies, soil, or plant-associated substrates, whereas hypocrealean fungi from humus substrates accumulated within tree holes remain poorly investigated. Tree-hole humus represents a spatially restricted and nutrient-rich microhabitat formed by decomposed plant material and other organic residues, and may harbor previously undocumented fungal taxa. In this study, hypocrealean fungi were investigated from humus substrates collected from tree holes in Doupengshan Scenic Area, Duyun City, Guizhou Province. Based on morphological observations and multi-locus phylogenetic analyses, two new species belonging to *Cordycipitaceae* and *Clavicipitaceae* were identified. These findings contribute to the knowledge of hypocrealean fungal diversity in karst microhabitats and provide additional taxonomic data for *Leptobacillium* and *Metapochonia*.

## Materials and methods

### Material collection and isolation

Humus substrates were collected from tree holes in Doupengshan Scenic Area, Duyun City, Qiannan Buyi and Miao Autonomous Prefecture, Guizhou Province, China, in October 2024 and October 2025. The samples were placed in sterile plastic bags and transported to the laboratory at 4 °C. For fungal isolation, approximately 1 g of each humus sample was suspended in 9 mL of sterile distilled water and shaken thoroughly to obtain a humus suspension. The suspension was serially diluted, and aliquots of the appropriate dilutions were spread onto potato dextrose agar (PDA) plates supplemented with 0.1 g/L streptomycin and 0.05 g/L tetracycline using a sterile spreader. The plates were incubated at 25 °C in the dark for 14 days. Emerging fungal colonies were selected based on colony morphology and transferred onto fresh PDA plates. Pure cultures were obtained by repeated subculturing of hyphal tips onto fresh PDA plates. To prepare holotypes, the pure cultures were dried at 50 °C and deposited in the Institute of Fungus Resources, Guizhou University (GZUIFR) (Zhang et al. 2023b). Living cultures were preserved on PDA slants at 4 °C and in 30% glycerol at -80 °C.

### Morphological characterization

The cultures were transferred to PDA and incubated at 25 °C for 14 days. Colony diameters and macromorphological characteristics were recorded, and photographs of the colony surface and reverse sides were taken using a Nikon D7500 camera (Nikon, Tokyo, Japan). To observe the micromorphological characteristics, hyphal fragments were mounted in sterile water. Morphological features, including conidiophores, phialides, and conidia, were examined using a light microscope (Axio Imager 2 Pol, ZEISS, Germany).

### DNA extraction, PCR amplification and sequencing

Genomic DNA was extracted using the Solarbio Fungal Genomic DNA Extraction Kit (Solarbio, D2300, China) and stored at -20 °C. Genetic markers were amplified by polymerase chain reaction (PCR). The primer pair ITS1/ITS4 was used to amplify the internal transcribed spacer (ITS) region (White et al. 1990), NS1/NS4 was used to amplify the small subunit ribosomal RNA (nrSSU) (White et al. 1990), LR0R/LR5 was used to amplify the large subunit ribosomal RNA (nrLSU) (Vilgalys and Hester 1990; Rehner and Samuels 1994), 983F/2218R was used to amplify the translation elongation factor-1 alpha (*tef*-*1α*) gene (Rehner and Buckley 2005), and CRPB1/RPB1Cr was used to amplify the largest subunit of RNA polymerase II (*rpb1*) (Castlebury et al. 2004). Amplification products were verified using 1% agarose gel electrophoresis. Purified PCR products were sent to Tsingke Biotechnology Co., Ltd. (Chongqing, China) for sequencing. The sequences obtained in this study have been deposited in GenBank (Table 1).

**Table 1.** Information and GenBank accession numbers of the related species used for phylogenetic analyses in this study.

**Species**	**Strain**	**ITS**	**nrSSU**	**nrLSU**	***tef*-*1α***	***rpb1***	**Reference**
*Keithomyces acicularis*	JCM 33284^T^	LC435734	LC435738	LC435741	LC462188	NA	(Iwasaki et al. 2019)
*Keithomyces acicularis*	JCM 33285	LC463198	LC435739	LC435742	LC462189	NA	(Iwasaki et al. 2019)
*Keithomyces carneus*	CBS 399.59	MT078887	NA	MH869445	NA	NA	(Sung et al. 2007a)
*Keithomyces carneus*	CBS 239.32^T^	AY624171	EF468988	EF468843	EF468789	EF468894	(Sung et al. 2007a)
*Leptobacillium cavernicola*	LRMH C212	OM622523	OM628842	OM628781	OM654332	OM677781	(Leplat et al. 2022)
*Leptobacillium cavernicola*	LRMH C216	OM622524	OM628843	OM628782	OM654333	OM677782	(Leplat et al. 2022)
*Leptobacillium cavernicola*	LRMH C217	MF788211	OM628844	OM628783	OM654334	OM677783	(Leplat et al. 2022)
*Leptobacillium chinense*	CGMCC 3.14969	JQ410323	NA	JQ410321	NA	NA	(Okane et al. 2020)
*Leptobacillium chinense*	CGMCC 3.14970^T^	JQ410324	NA	JQ410322	NA	NA	(Okane et al. 2020)
*Leptobacillium coffeanum*	COAD 2057	MF066034	NA	MF066032	NA	NA	(Okane et al. 2020)
*Leptobacillium coffeanum*	COAD 2061	MF066035	NA	MF066033	NA	NA	(Okane et al. 2020)
***Leptobacillium doupengense***	**GZUIFR DPS001^T^**	**PX844695**	**PX844697**	**PX844704**	**PX893664**	**PX893665**	**This study**
***Leptobacillium doupengense***	**GZUIFR DPS003**	**PX845559**	**PX845586**	**PX845587**	**PX893666**	**PX893667**	**This study**
*Leptobacillium filiforme*	URM 7918^T^	NA	NA	MH979399	NA	NA	(Chen et al. 2021)
*Leptobacillium latisporum*	TBRC 16288^T^	OP856540	OP850838	OP856529	NA	NA	(Preedanon et al. 2023)
*Leptobacillium leptobactrum*	ZJ14B02	PP385689	NA	PP381743	NA	NA	(Zare and Gams 2016)
*Leptobacillium leptobactrum*	AH17C05	PP384754	NA	PP380808	NA	NA	(Zare and Gams 2016)
Leptobacillium leptobactrum var. calidius	CBS 703.86	EF641866	EF641850	KU382226	NA	NA	(Zare and Gams 2016)
Leptobacillium leptobactrum var. leptobactrum	CBS 771.69	EF641868	EF641852	KU382224	NA	NA	(Zare and Gams 2016)
*Leptobacillium longiphialidum*	YFCC 23039272	PQ509282	PQ508806	PQ508808	PQ560997	PQ567240	(Lu et al. 2025)
*Leptobacillium longiphialidum*	YFCC 24079491	PQ509281	PQ508805	PQ508807	PQ560996	PQ567239	(Lu et al. 2025)
*Leptobacillium marksiae*	BRIP 70307a^T^	PQ061114	NA	PQ047739	NA	NA	(Tan and Shivas 2024)
*Leptobacillium muralicola*	CGMCC 3.19014	MH379983	NA	MH379997	NA	NA	(Sun et al. 2019)
*Leptobacillium muralicola*	CGMCC 3.19015	MH379985	NA	MH379999	NA	NA	(Sun et al. 2019)
*Leptobacillium symbioticum*	NBRC 113865^T^	LC485673	NA	LC506046	NA	NA	(Okane et al. 2020)
*Leptobacillium xianyushanense*	RCEF6793^T^	OQ780699	NA	OQ780702	NA	NA	(Chang et al. 2024)
*Leptobacillium xianyushanense*	RCEF6795	OQ780930	NA	OQ780931	NA	NA	(Chang et al. 2024)
*Marquandomyces marquandii*	CBS 182.27^T^	MH854923	EF468990	MH866418	EF468793	EF468899	(Sung et al. 2007a)
*Marquandomyces marquandii*	CBS 128893	MH865143	NA	MH876582	NA	NA	(Sung et al. 2007a)
*Marquandomyces* sp.	CBS 127132	MT078882	MT078872	MH875873	MT078849	MT078865	(Sung et al. 2007a)
*Marquandomyces* sp.	CBS 129413	NA	MT078874	MH876751	MT078851	MT078867	(Sung et al. 2007a)
*Metapochonia bulbillosa*	CBS 145.70^T^	MH859529	AF339591	AF339542	EF468796	EF468902	(Kepler et al. 2014)
*Metapochonia cordycipiticonsociata*	CGMCC 317366^T^	KM263567	KM263570	KM263574	KM263582	KM263578	(Labuda et al. 2018)
*Metapochonia cordycipiticonsociata*	CGMCC 317367	KM263568	KM263571	KM263575	KM263583	KM263577	(Labuda et al. 2018)
***Metapochonia doupengshanensis***	**GZUIFR DPS110^T^**	**PX849742**	**PX849745**	**PX849746**	**PX893668**	**PX893669**	**This study**
***Metapochonia doupengshanensis***	**GZUIFR DPS111**	**PX849747**	**PX849748**	**PX849749**	**PX893670**	**PX893671**	**This study**
*Metapochonia goniodes*	CBS 891.72^T^	MH860626	AF339599	MH872319	DQ522354	DQ522401	(Kepler et al. 2014)
*Metapochonia hahajimaensis*	JCM 34019^T^	LC529951	LC529955	LC529959	LC529963	NA	(Kondo et al. 2020)
*Metapochonia lutea*	BiMM-F96/DF4^T^	MF983717	NA	NA	MF983718	NA	(Labuda et al. 2018)
*Metapochonia microbactrospora*	CBS 101433^T^	NA	NA	MH874343	KJ398794	NA	(Kepler et al. 2014)
*Metapochonia parasitica*	ARSEF 3436	FJ973068	EF468993	EF468848	EF468799	EF468904	(Barron 1980)
*Metapochonia rubescens*	CBS 464.88^T^	MH862138	NA	AF339566	EF468797	EF468903	(Kepler et al. 2014)
*Metapochonia simonovicovae*	BiMMF299^T^	PP085431	NA	PP085432	PP085550	NA	(Crous et al. 2024)
Metapochonia suchlasporia var. catenata	CBS 248.83^T^	MH861579	NA	MH873310	KJ398789	NA	(Kepler et al. 2014)
Metapochonia suchlasporia var. suchlasporia	CBS 251.83^T^	MH861580	NA	MH873311	KJ398790	NA	(Kepler et al. 2014)
*Metapochonia variabilis*	CGMCC 3.17925^T^	NA	KY883278	NA	NA	KY883214	(Zhang et al. 2017)
*Metapochonia variabilis*	CGMCC 3.17926	NA	KY883279	NA	NA	KY883215	(Zhang et al. 2017)
*Metarhizium anisopliae*	BUM_1900	NA	NA	NA	MH143854	MH143869	(Zimmermann 1993)
*Metarhizium flavoviride*	CBS 125.65	MT078885	MT078869	MT078854	MT078846	MT078862	(Kepler et al. 2014)
*Metarhizium flavoviride*	CBS 700.74	NA	MT078870	MT078855	MT078847	MT078863	(Kepler et al. 2014)
*Papiliomyces longiclavatus*	YC 20061403	MZ702080	MZ702112	MZ702101	MZ955880	MZ955880	(Zhang et al. 2023a)
*Papiliomyces sinensis*	GMBC 3053^T^	PQ636503	PQ636500	PQ636506	PQ660655	PQ660658	(Chen et al. 2025)
*Papiliomyces sinensis*	GMBC 3054	PQ636504	PQ636501	PQ636507	PQ660656	PQ660659	(Chen et al. 2025)
*Pochonia chlamydosporia*	CBS 101244	DQ522544	DQ518758	DQ518758	DQ522327	DQ522372	(Gams and Zare 2001)
Pochonia chlamydosporia var. catenulata	JCM 18598	AB758248	AB709810	AB709810	AB758456	AB758659	(Gams and Zare 2001)
Pochonia chlamydosporia var. catenulata	JCM 18600	AB758266	AB709812	AB709812	AB758474	AB758677	(Gams and Zare 2001)
Pochonia chlamydosporia var. chlamydosporia	CM 18605	AB758261	AB709817	AB709817	AB758469	AB758672	(Gams and Zare 2001)
Pochonia chlamydosporia var. chlamydosporia	JCM 18607	AB758270	AB709819	AB709819	AB758478	AB758681	(Gams and Zare 2001)
Pochonia chlamydosporia var. ellipsospora	JCM 18609^T^	AB758257	AB709821	AB709821	AB758465	AB758668	(Nonaka et al. 2013b)
Pochonia chlamydosporia var. ellipsospora	JCM 18611	AB758265	AB709823	AB709823	AB758473	AB758676	(Nonaka et al. 2013b)
Pochonia chlamydosporia var. spinulospora	JCM 18613^T^	AB758258	AB709827	AB709827	AB758466	AB758669	(Nonaka et al. 2013b)
Pochonia chlamydosporia var. spinulospora	JCM 18619	AB758272	AB709830	AB709830	AB758480	AB758683	(Nonaka et al. 2013b)
*Pochonia sinensis*	ZY 22.009^T^	OQ709251	OQ709263	OQ709263	OQ719629	NA	(Zhang et al. 2023b)
*Pochonia sinensis*	ZY 22.010	OQ709252	OQ709264	OQ709264	OQ719630	NA	(Zhang et al. 2023b)
*Samsoniella anhuiensis*	RCEF2830	OM268837	OM268843	OM268848	OM483864	OM751889	(Wang et al. 2024)
*Samsoniella anhuiensis*	RCEF2590	NA	OR978313	OR978316	OR966516	OR989964	(Wang et al. 2024)
*Samsoniella antleroides*	YFCC 6016	OQ476471	MN576747	MN576803	OQ506162	MN576863	(Wang et al. 2020)
*Samsoniella antleroides*	YFCC 6113	OQ476472	MN576748	MN576804	OQ506163	MN576864	(Wang et al. 2020)
*Simplicillium aogashimaense*	JCM 18167^T^	AB604002	NA	LC496874	LC496904	NA	(Nonaka et al. 2013a)
*Simplicillium aogashimaense*	JCM 18168	AB604004	NA	LC496875	NA	NA	(Nonaka et al. 2013a)
*Simplicillium calcicola*	LC5586^T^	KU746706	NA	KU746752	KX855252	NA	(Zhang et al. 2017)
*Simplicillium calcicola*	LC5371	KU746705	NA	KU746751	KX855251	NA	(Zhang et al. 2017)
*Simplicillium lamellicola*	JC-1	MT807906	MT807908	MT807907	NA	NA	(Zare and Gams 2001)
*Simplicillium lamellicola*	CBS 116.25^T^	AJ292393	NA	NA	DQ522356	DQ522404	(Zare and Gams 2001)
*Simplicillium minatense*	JCM 18176^T^	AB603992	LC496893	LC496878	LC496908	NA	(Nonaka et al. 2013a)
*Simplicillium minatense*	JCM 18178	AB603993	LC496894	LC496879	LC496909	NA	(Nonaka et al. 2013a)
*Simplicillium obclavatum*	CBS 311.74^T^	AJ292394	NA	AF339517	EF468798	NA	(Gams 2017)
*Simplicillium obclavatum*	SUF81	NA	NA	MK788174	NA	NA	(Gams 2017)
*Simplicillium scarabaeoidea*	DY101391	MT453842	MT453843	NA	MT471335	MT471343	(Chen et al. 2021)
*Simplicillium scarabaeoidea*	DY101392	MT453845	NA	NA	MT471336	NA	(Chen et al. 2021)
*Simplicillium subtropicum*	JCM 18180^T^	AB603990	NA	LC496880	LC496910	NA	(Nonaka et al. 2013a)
*Simplicillium subtropicum*	JCM 18181	AB603995	NA	LC496881	LC496911	NA	(Nonaka et al. 2013a)
*Simplicillium sympodiophorum*	JCM 18184^T^	AB604003	NA	LC496882	LC496912	NA	(Nonaka et al. 2013a)
*Sungia yongmunensis*	EFCC 2131^T^	JN049856	EF468977	EF468833	EF468770	EF468876	(Sung et al. 2007b)
*Sungia yongmunensis*	EFCC 2135	NA	EF468979	EF468834	EF468769	EF468877	(Sung et al. 2007b)
*Yosiokobayasia kusanagiensis*	TNS F18494	JN049873	JF415954	JF415972	JF416014	JN049890	(Kepler et al. 2012)
*Yosiokobayasia kusanagiensis*	BUM 1307	OM955151	OM951246	OM951251	OM988199	NA	(Sung et al. 2007a)

### Phylogenetic analyses

The DNA sequences generated in this study were assembled and edited using DNASTAR Lasergene (v. 6.0). Sequences of five loci (ITS, nrSSU, nrLSU, *tef*-*1α* and *rpb1*) retrieved from GenBank, together with the newly obtained sequences, were aligned and trimmed using MEGA (v. 6.06) (Tamura et al. 2013). PhyloSuite (v. 2) was used for concatenation and analysis of the combined dataset (Zhao et al. 2025). The optimal nucleotide substitution model for each gene partition was selected based on the corrected Akaike Information Criterion (AICc) using ModelFinder (v. 3.0.1) (Kalyaanamoorthy et al. 2017).

Phylogenetic analyses of the concatenated loci were performed using both Bayesian inference (BI) and maximum likelihood (ML) methods. BI was conducted using MrBayes v. 3.2.7a to infer phylogenetic trees from the combined datasets (Ronquist et al. 2012). The Markov chain Monte Carlo (MCMC) analysis was run for 10 million generations, sampling every 500 generations, resulting in 20,000 trees. The first 25% of trees were discarded as burn-in, and the remaining trees were used to calculate posterior probabilities for the majority-rule consensus tree. Maximum likelihood analysis was performed using IQ-TREE v. 3.0.1 (Nguyen et al. 2015). Branch support was assessed using the ultrafast bootstrap method with 5,000 replicates (Minh et al. 2013). The resulting phylogenetic trees were visualized using the Interactive Tree of Life (iTOL) v. 6 (Letunic and Bork 2016).

## Results

### Phylogenetic analyses

**Figure 1.**
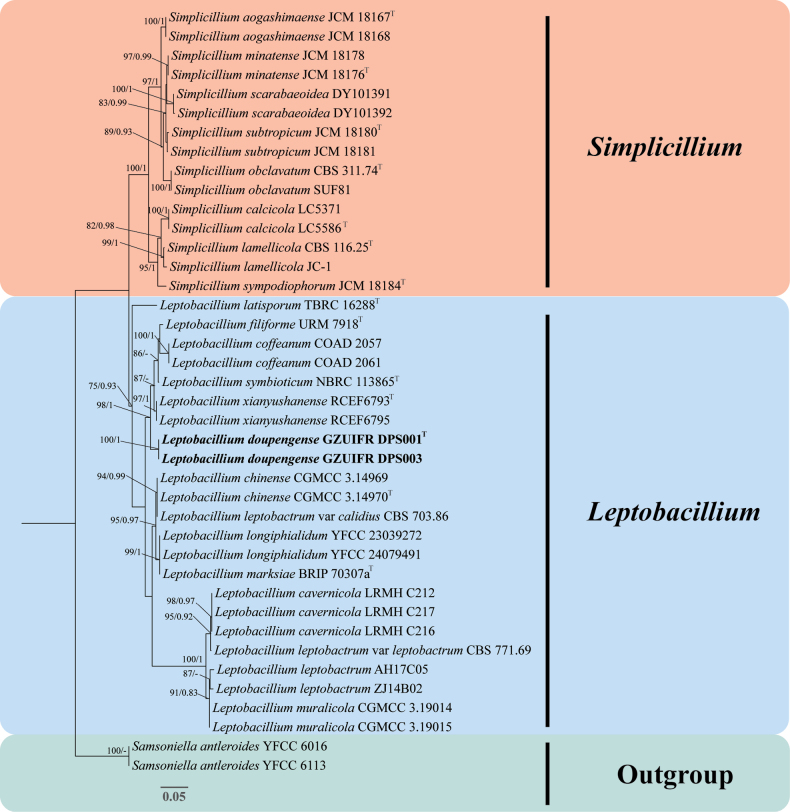
Phylogenetic analysis of the new strains and their related species based on a multi-locus sequence dataset (ITS, nrSSU, nrLSU, *tef*-*1α* and *rpb1*). Statistical support values (≥75% ML / 0.75 PP) are indicated at the nodes, representing maximum likelihood bootstrap support and Bayesian inference posterior probabilities, respectively. Isolates obtained in this study are shown in bold.

**Figure 2.**
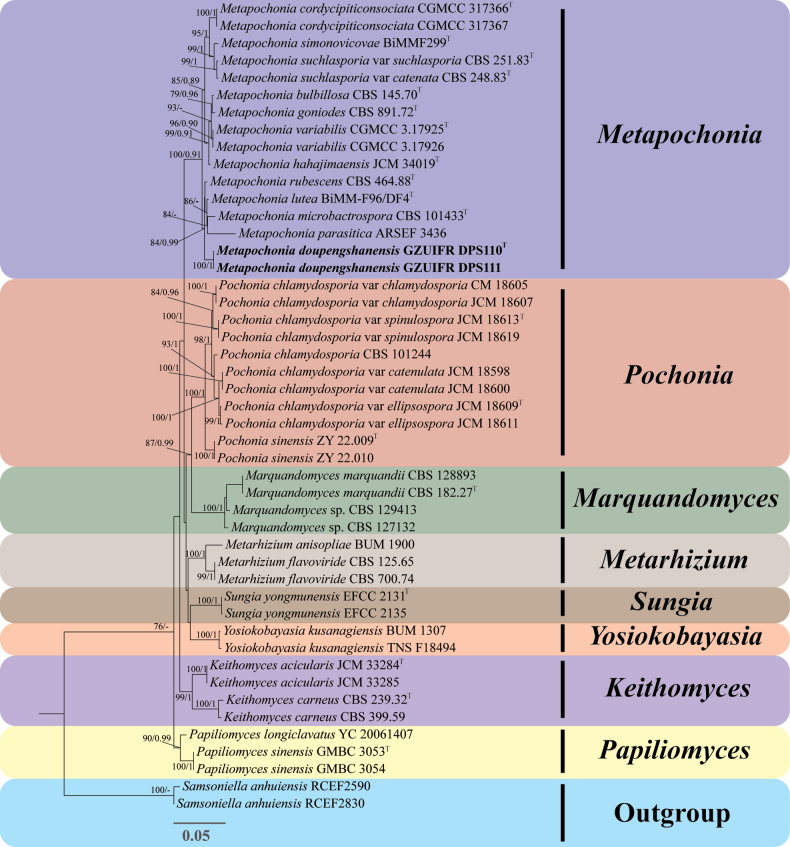
Phylogenetic analysis of the new strains and their related species based on a multi-locus sequence dataset (ITS, nrSSU, nrLSU, *tef*-*1α* and *rpb1*). Statistical support values (≥75% ML / 0.75 PP) are shown at the nodes, representing maximum likelihood bootstrap support and Bayesian inference posterior probabilities, respectively. Isolates obtained in this study are shown in bold.

Phylogenetic trees were constructed based on five gene loci (ITS, nrSSU, nrLSU, *tef*-*1α* and *rpb1*). The trees generated by ML and BI analyses (Figs 1, 2) were largely congruent in topology. These results are consistent with the established phylogenetic framework of related hypocrealean fungi (Kondo et al. 2020; Chang et al. 2024; Crous et al. 2024; Lu et al. 2025).

In the phylogenetic tree of *Leptobacillium* (Fig. 1), *Samsoniella antleroides* (YFCC 6016 and YFCC 6113) was selected as the outgroup. The combined dataset comprised 40 sequences and 1,449 characters. The optimal model for maximum likelihood analysis was TN+I+G4, and the log-likelihood value of the best-scoring tree was -5310.456. The parameters of the GTR model were estimated as follows: substitution rates AC = 1.00000, AG = 1.68881, AT = 1.00000, GC = 1.00000, CT = 4.84415, GT = 1.00000. The optimal model for Bayesian inference was GTR+I+G. The strains GZUIFR DPS001 and GZUIFR DPS003 clustered together and formed an independent lineage within *Leptobacillium* with strong support (100% ML / 1 PP), phylogenetically close to but clearly separated from *L. xianyushanense*.

In the phylogenetic analysis of *Metapochonia* (Fig. 2), *Samsoniella anhuiensis* (RCEF 2590 and RCEF 2830) was selected as the outgroup. The combined dataset comprised 47 sequences and 3,801 characters. Based on the multi-locus phylogenetic analysis, the optimal model for maximum likelihood was TIM2+R2, and the log-likelihood value of the best-scoring tree was -13350.881. The parameters of the GTR model were estimated as follows: substitution rates AC = 1.50683, AG = 2.65597, AT = 1.50683, GC = 1.00000, CT = 7.51043, GT = 1.00000. The optimal model for Bayesian inference was GTR+I+G4. The two strains GZUIFR DPS110 and GZUIFR DPS111 clustered together and formed a distinct, well-supported lineage within *Metapochonia* (100% ML / 1 PP). This lineage was phylogenetically close to *M. rubescens* (CBS 464.88) but clearly separated from it and other related species in the genus.

### Taxonomy

**Kingdom *Fungi***

**Division *Ascomycota***

**Order *Hypocreales***

**Family *Cordycipitaceae***

**Genus *Leptobacillium***

#### Leptobacillium doupengense

Taxon classificationFungiHypocrealesCordycipitaceae

W.K. Lin, Y.M. Zhou & L.L. Lou sp. nov.

6506C2DE-A039-5E31-89FB-E81B14D37B57

862246

Fig. 3

##### Etymology.

The specific epithet refers to Doupengshan Scenic Area, the locality where the species was collected.

**Figure 3.**
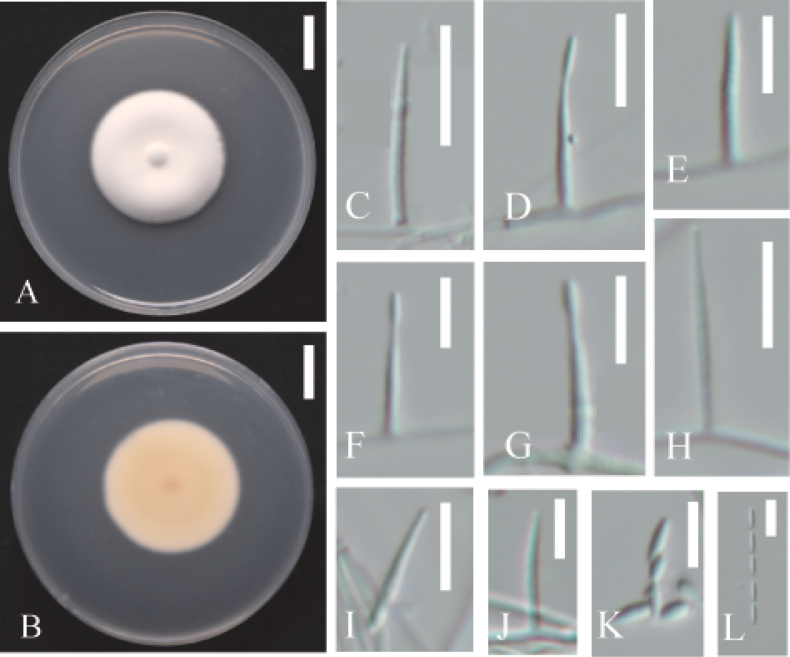
Morphology of *Leptobacillium doupengense* on PDA after 14 days of growth at 25 °C. **A, B**. Front and reverse colony views on PDA; **C–J**. Phialides; **K, L**. Conidia. Scale bars: 10 mm (**A, B**); 10 μm (**C–L**).

##### Type.

China, • Guizhou Province, Qiannan Buyi and Miao Autonomous Prefecture, Duyun City, Doupengshan Scenic Area (26°22'17"N, 107°23'31"E), from humus substrates accumulated within tree holes, October 2024, collected by Wen-Kai Lin (holotype: GZUIFR DPS001, dried culture specimen; ex-type culture: GZUIFR DPS003, derived from the holotype material).

##### Description.

**Sexual morph**: Not observed. **Asexual morph**: On PDA after 14 days at 25 °C, colonies reached 35–40 mm in diameter. Colony surface white, raised, circular with entire margin, mycelium velvety to floccose, reverse smooth, pale yellow, orange-yellow to cream-yellow, with yellow pigment diffusing into the agar. Hyphae septate, hyaline, smooth-walled, 1–2 μm wide. Conidiophores arising from aerial hyphae, hyaline, septate, smooth-walled. Phialides mostly solitary, rarely verticillate, borne terminally on conidiophores or lateral branches, 15–23 × 1.8–2.2 μm (n = 30), tapering toward the apex. Conidia formed in slender chains, hyaline, fusiform to narrowly cylindrical, 3–4 × 0.5–1 μm (mean = 3.5 × 1.0 μm, n = 30). Chlamydospores absent.

##### Habitat.

On humus substrates collected from tree holes of unidentified tree species.

##### Distribution.

China, Guizhou Province, Qiannan Buyi and Miao Autonomous Prefecture, Duyun City.

##### Notes.

Phylogenetic analyses based on multi-locus sequence data revealed that *Leptobacillium doupengense* forms a distinct and well-supported clade. Phylogenetically, *L. doupengense* is placed in a subclade with *L. coffeanum*, *L. symbioticum*, *L. filiforme*, and *L. xianyushanense*. Pairwise sequence comparisons in the ITS region revealed that *L. doupengense* differs from *L. coffeanum* (COAD 2057) by 28 bp with 4 gaps (93.9% similarity), from *L. filiforme* (URM 7918) by 25 bp with 3 gaps (94.6%), from *L. symbioticum* (NBRC 113865) by 22 bp with 1 gap (95.2%), and from *L. xianyushanense* (RCEF 6793) by 15 bp with 1 gap (96.7%). Species delimitation in this study was based on an integrative taxonomic approach, including an independent and well-supported placement in the multi-locus phylogeny, ITS sequence divergence from closely related species, and diagnosable morphological differences.

Morphologically, *L. doupengense* is similar to most species of *Leptobacillium* in producing long, slender chains of conidia. However, it can be distinguished from its closely related species by several morphological characteristics. Compared with *L. coffeanum*, *L. doupengense* produces conidia arranged in chains, whereas the conidia of *L. coffeanum* are produced in slimy heads (Gomes et al. 2018). Although *L. doupengense* is morphologically similar to *L. xianyushanense*, it differs from the latter in colony growth and colony surface morphology. On PDA at 25 °C after 14 days, colonies of *L. doupengense* reached 35–40 mm in diameter and were slightly raised, smooth, and circular, without obvious divergent cracks. In contrast, colonies of *L. xianyushanense* grow more slowly, reaching 20–36 mm in diameter after 14 days at 25 °C, and have a white, irregular floccose surface characterized by divergent cracks. Additionally, *L. doupengense* tends to produce shorter conidia (3–4 × 0.5–1 μm) than *L. symbioticum* (4.0–6.9 × 0.7–1.6 μm), *L. filiforme* (7.2–12.5 × 1.0 μm), and *L. xianyushanense* (3.8–5.6 × 0.5–1.2 μm) (Okane et al. 2020; Chen et al. 2021; Chang et al. 2024). Therefore, the combination of colony morphology, conidial size, ITS sequence divergence, and independent phylogenetic placement supports the recognition of *L. doupengense* as a distinct species within *Leptobacillium*.

###### Family *Clavicipitaceae*

**Genus *Metapochonia***

#### Metapochonia doupengshanensis

Taxon classificationFungiHypocrealesClavicipitaceae

W.K. Lin, Y.M. Zhou & L.L. Lou sp. nov.

8804837B-5693-59AB-AFDE-79E3A39B10FB

862247

Fig. 4

##### Etymology.

The specific epithet refers to Doupengshan Scenic Area, the locality where the species was collected.

**Figure 4.**
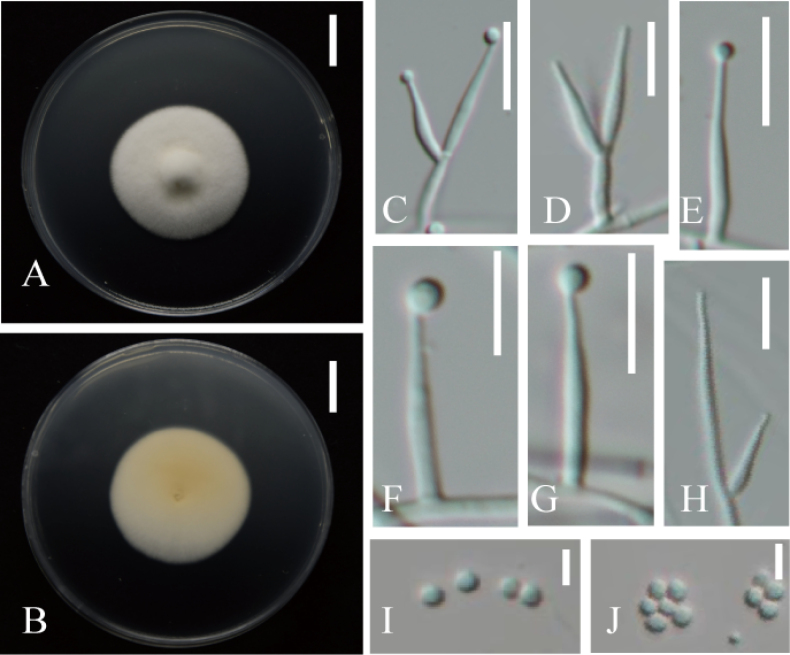
Morphology of *Metapochonia doupengshanensis* on PDA after 14 days of growth at 25 °C. **A, B**. Front and reverse colony views on PDA; **C–H**. Phialides; **I, J**. Conidia. Scale bars: 10 mm (**A, B**); 10 μm (**C–J**).

**Table 2.** Morphological comparison of the new species with closely related species in the genus *Leptobacillium*.

**Species**	**Phialides**	**Conidia**	**Substrate**	**References**
*L. doupengense*	Predominantly solitary, rarely verticillate, tapering from the base to the apex. 15–23 × 1.8–2.2 μm	Fusiform, narrowly cylindrical. 3–4 × 0.5–1 μm	humus substrate within tree holes	This study
*L. coffeanum*	Predominantly solitary, rarely in whorls of 2–3. 11–44 (–70) × 1.0–2.4 μm	Two types of conidia: larger, 5.3–8.8 × 1.0–1.6 μm; smaller, 2.2–3.8 × 0.8–1.5 μm.	Coffee branches	Gomes et al. (2018)
*L. symbioticum*	Predominantly solitary, rarely branched. Slender, tapering toward the apex. 7.1–30.6 × 1.6–3.5 μm	Slightly fusiform to narrowly cylindrical. 4.0–6.9 × 0.7–1.6 μm	Prostigmata, sori of *Phakopsora pachyrhizi*	Okane et al. (2020)
*L. filiforme*	Solitary, gradually tapering toward the apex. 9–18 × 1 μm	Fusiform to filiform. 7.2–12.5 × 1.0 μm	*Citrullus lanatus*	Crous et al. (2018)
*L. xianyushanense*	Phialides are predominantly solitary, gradually tapering toward the apex. 24.7–28.5 × 1.5–1.9 μm	Narrowly cylindrical to slightly fusiform. 3.8–5.6 × 0.5–1.2 μm	Rhizosphere of *Camellia oleifera*	Chang et al. (2024)

**Table 3.** Morphological comparison of the new species with closely related species in the genus *Metapochonia*.

**Species**	**Phialides**	**Conidia**	**Conidial mass**	**References**
*M. doupengshanensis*	15–24.5 × 1–2.5 μm	Globose to ellipsoidal. 2–2.5 × 1.5–2 μm	Conidia produced in slimy heads.	This study
*M. lutea*	22–25 × 1.5 μm	The first type is ellipsoidal to ovoid. 3.0–3.5 × 2.0 μm The second type is slightly curved or reniform. 3.5 × 2.5 μm	Spherical pseudocapitate structures.	Labuda et al. (2018)
*M. microbactrospora*	–	Ellipsoidal to bacilliform. 2–2.5 × 0.7–1.0 μm	Slimy spore heads.	Zare et al. (2001)
*M. parasitica*	8.5–15 × 7–10 μm	Spherical to subglobose. 3.0–4.5 × 2.5–3.0 μm	–	Barron (1980)
*M. rubescens*	18–25 × 0.7–1.0 μm	Spherical to subglobose. 2.5–3.5 × 2.0–3.0 μm	Dry spore heads.	Zare et al. (2001)

##### Type.

China, • Guizhou Province, Qiannan Buyi and Miao Autonomous Prefecture, Duyun City, Doupengshan Scenic Area (26°21'31"N, 107°25'16"E), from humus substrates accumulated within tree holes, 23 October 2025, collected by Li-Li Lou (holotype: GZUIFR DPS110, dried culture specimen; ex-type culture: GZUIFR DPS111, derived from the holotype material).

##### Description.

**Sexual morph**: Not observed. **Asexual morph**: On PDA after 14 days at 25 °C, colonies were slow-growing, reaching 38–41 mm in diameter, surface white, floccose, subcircular, with an entire margin and raised center; reverse smooth, orange-yellow to yellow. Hyphae hyaline, smooth-walled, branched, septate, 1–2 μm wide. Conidiophores arising from aerial hyphae, mostly prostrate, rarely erect, hyaline, smooth-walled. Phialides solitary or occasionally in pairs, slender, with a cylindrical basal portion, tapering toward the apex, 15–24.5 × 1–2.5 μm (n = 30). Conidia produced at the tips of phialides, solitary or in globose to subglobose heads, unicellular, hyaline, globose to ellipsoidal, 2–2.5 × 1.5–2 μm (mean = 2.0 × 1.5 μm, n = 30). Chlamydospores and crystals not observed.

##### Habitat.

On humus substrates collected from tree holes of unidentified tree species.

##### Distribution.

China, Guizhou Province, Qiannan Buyi and Miao Autonomous Prefecture, Duyun City.

##### Notes.

Phylogenetic analyses based on multi-locus sequence data revealed that *Metapochonia doupengshanensis* forms a distinct and well-supported clade within the genus *Metapochonia* (100% ML / 1 PP). It is phylogenetically placed in a subclade together with *M. lutea*, *M. microbactrospora*, *M. parasitica*, and *M. rubescens*, indicating a close relationship with this group of species. Morphologically, the genus *Metapochonia* is characterized by producing conidia on slender phialides, which may be verticillate or solitary, and most species are also known to produce chlamydospores. *Metapochonia doupengshanensis* is morphologically most similar to *M. rubescens*, but can be distinguished by its globose to ellipsoidal conidia (2–2.5 × 1.5–2 μm), whereas *M. rubescens* produces globose to subglobose conidia (2.5–3.5 × 2.0–3.0 μm) (Zare et al. 2001). Additionally, pairwise sequence analysis of the *tef*-*1α* gene revealed that *M. doupengshanensis* differs from its close relatives by 11–25 bp with 1–3 gaps. Specifically, it showed 98.8% similarity with *M. rubescens* (11 bp, 3 gaps), 98.6% with *M. lutea* (13 bp, 1 gap), 97.8% with *M. microbactrospora* (20 bp, 3 gaps), and 97.4% with *M. parasitica* (25 bp, 1 gap). Species delimitation in this study was based on an integrative taxonomic approach, including an independent and well-supported placement in the multi-locus phylogeny, *tef*-*1α* sequence divergence from closely related species, and diagnosable morphological differences. Therefore, both morphological and molecular data support the recognition of *M. doupengshanensis* as a new species within *Metapochonia*.

## Discussion

Sequence data for several loci of *Leptobacillium*, such as nuclear ribosomal small subunit DNA (nrSSU), the translation elongation factor 1-α gene (*tef*-*1α*), and the largest subunit of RNA polymerase II (*rpb1*), remain limited in public databases. This lack of data may restrict the resolution of phylogenetic relationships within *Leptobacillium* and between this genus and other closely related genera in *Cordycipitaceae*. Therefore, the accumulation of multi-locus sequence data from newly collected taxa is important for improving species delimitation and phylogenetic resolution within the genus. The recently reported taxon *Leptobacillium hepiali* X. Zou, J. Shen & G.X. Fei from Guizhou Province was also considered because of its geographical relevance to the present study. However, its nomenclatural status appears to be uncertain, and the available sequence data suggest that it is not positioned within the clade containing *L. doupengense* and its closely related species. Therefore, it was not treated as a key comparative taxon in the present study.

Several species closely related to *L. doupengense*, including *L. coffeanum*, *L. symbioticum*, *L. filiforme*, and *L. xianyushanense*, have been reported from plants or plant-derived substrates (Gomes et al. 2018; Okane et al. 2020; Chang et al. 2024). In the present study, *L. doupengense* was isolated from humus accumulated within a tree hole, a substrate mainly derived from decomposed plant material. These observations suggest a possible ecological association between this lineage and plant-derived organic substrates. Given that tree-hole humus is mainly composed of decomposed plant material, *L. doupengense* may be involved in the colonization or turnover of plant-derived organic matter in this microhabitat, although this ecological role requires further experimental confirmation. However, because only a limited number of isolates are currently available and no ecological experiments were conducted, this possible substrate association should be interpreted cautiously. Further sampling from different substrates and habitats is needed to clarify the ecological preferences of this lineage.

Unlike the substrate associations observed in *Leptobacillium*, species of *Metapochonia* are commonly found on animal-derived substrates, such as nematode eggs or cysts (Kepler et al. 2014). In this study, *M. doupengshanensis* was isolated from tree-hole humus rather than directly from animal-derived substrates. This finding expands the known substrate range of *Metapochonia* and suggests that tree-hole humus may provide suitable microhabitats for hypocrealean fungi with different ecological affinities. Considering that some members of *Metapochonia* are associated with nematode eggs or cysts, the occurrence of *M. doupengshanensis* in tree-hole humus may also reflect potential interactions with microfauna or organic-matter-associated microbial communities; however, this hypothesis remains to be tested, and the ecological role of this species in tree-hole humus remains unknown.

The karst region of Guizhou Province, China, is characterized by complex topography, heterogeneous microhabitats, and rich fungal diversity. Numerous species of *Hypocreales* have been discovered recently in the region (Chen et al. 2021, 2022a, 2022b, 2022c, 2022d, 2025; Bu et al. 2025; Xu et al. 2025). Tree holes in karst forests often accumulate humus and represent spatially discrete, relatively stable, and nutrient-rich microhabitats. However, hypocrealean fungi in such microhabitats remain poorly studied. The discovery of *L. doupengense* and *M. doupengshanensis* from tree-hole humus highlights the potential importance of this overlooked microhabitat as a reservoir of fungal diversity in karst ecosystems. Further surveys combining broader sampling, culture-dependent isolation, high-throughput sequencing, and ecological observations are needed to better understand the diversity and ecological functions of hypocrealean fungi in tree-hole humus.

## Supplementary Material

XML Treatment for Leptobacillium doupengense

XML Treatment for Metapochonia doupengshanensis

## Additional information

### Artificial Intelligence (AI) use

The authors accept full responsibility for the content of the manuscript, including the disclosure of any use of AI.

Artificial intelligence (AI) tools were used only for language polishing during manuscript preparation. No AI tools were used for image processing, data analysis, phylogenetic analysis, taxonomic decision-making, or scientific interpretation. The authors reviewed and edited all AI-assisted text and take full responsibility for the content of the manuscript.

### Author ORCIDs

W.-K. Lin https://orcid.org/0009-0007-3198-7986

L.-L. Lou https://orcid.org/0009-0000-1072-3120

X. Zou https://orcid.org/0000-0002-3666-5536

Y.-M. Zhou https://orcid.org/0000-0001-8563-8000

### Data availability

All of the data that support the findings of this study are available in the main text.
